# Management of Quadruplet Pregnancy: A Case Report

**DOI:** 10.31729/jnma.4861

**Published:** 2020-02-29

**Authors:** Lhakpa Dolma Lama, Tanya Das, Asmita Neupane, Roshan Lama, Rakshya Pandey, Urmila Karki

**Affiliations:** 1 Department of Gynecology and Obstetrics, Kathmandu Medical College and Teaching Hospital, Sinamangal, Kathmandu, Nepal; 2 Kathmandu Medical College and Teaching Hospital, Sinamangal, Kathmandu, Nepal; 3 Deurali Primary Health Center, Nuwakot, Nepal

**Keywords:** *case report*, *cesarean section*, *fertility agent*, *multiple pregnancy*, *pregnancy*, *quadruplets*

## Abstract

Quadruplet pregnancy is a pregnancy state where four fetuses grow simultaneously inside a mother’s womb. Four fetuses developing in a womb is a challenge not only to the mother but to the obstetrician who has to calculate every risk associated with such pregnancy. High order pregnancy is considered a high risk pregnancy due to increase in maternal, fetal and neonatal morbidity and mortality. So a multidisciplinary approach with early involvement of neonatologists and anesthesiologists for the assessment of such case is essential for a successful obstetric outcome. Here we present a case report of 27 years G_3_P_1_L_1_A_1_ at 33 weeks 2 days of gestation with quadruplet pregnancy with previous lower segment Cesarian section with history of ovulation induction, delivered successfully via cesarean section with successful outcome of all 1 female and 3 male babies.

## INTRODUCTION

Higher order pregnancy is usually considered a high risk pregnancy due to associated increase in maternal, fetal and neonatal morbidity and mortality.^1-4^ The incidence of such higher order multiple pregnancies ranges from 0.01% to 0.07% of all the pregnancies.^5^ However, the frequency of multiple gestations has been in a rising trend due to evolution of assisted reproductive techniques which defies the Hellin’s Law, that is, quadruplets occurring 1:80 which translates to 1 in 512,000 live births.^3^ Higher order multiple pregnancies could be monozygotic, multizygotic or a combination of the two.^6^ Here we present a case of quadruplet pregnancy in 27 years G_3_P_1_L_1_A_1_, delivered successfully at 33 weeks 2 days of gestation via caesarean section.

## CASE REPORT

A 27 years, G_3_P_1_L_1_A_1_ was presented and admitted at 32 weeks 1 day of gestation with the chief complaints of bilateral lower limb swelling for a duration of 1 month. She had been married for 8 years with previous 1 induced abortion and one female child aged 6 years who was delivered via cesarean section due to cephalo-pelvic disproportion. She was diagnosed as a case of quadruplet pregnancy with quadriamniotic quadrichorionic placenta by ultrasound done at 12 weeks of gestation. She also gives history of intake of medication for conception of this pregnancy for 1 cycle as she was unable to conceive for 1 year. However exact documentation was not available since they took the medication in a local clinic. Her previous menstrual cycles were regular and her marriage was a non-consanguineous one. There was no family history of multiple pregnancies. She registered for antenatal care at 17 weeks of gestation in our hospital during which she was started on hematinics and calcium from second trimester onward. Previous antenatal care was at a local clinic.

Baseline blood and urine investigations and serial ultrasonography was done. Anomaly scan at 20 weeks showed no obvious congenital anomalies in any of the four fetuses. She was vaccinated with 2 doses of tetanus diphtheria vaccine. Pregnancy was monitored by regular antenatal checkups. Ultrasonography done on 28 and 31 weeks of gestation were normal. The latter showed live quadruplet gestation (cephalic, oblique, transverse, transverse) compatible with stated gestational age. The estimated fetal weights ranged from 1.3-1.7kg. Two anterior and two lateral placentation at upper uterine segment in location and the liquor volume was normal.

At 32 weeks of gestation she was admitted with the above mentioned complain, her investigations were within normal limits. On examination, her abdomen was over distended with multiple fetal parts palapable. Four fetal heart sound were audible on auscultation. Vaginal examination revealed a tubular, closed and uneffaced cervix. She was continued on hematinics and calcium supplementation. However with each passing day she developed abdominal discomfort. During her hospital stay she received 4 doses of injection dexamethasone 6mg, 12 hours apart. On her 5^th^ day of admission, she developed sore throat and fever for which further investigations were done and Dengue NS1 Antigen was found positive, she was managed conservatively with IV fluids and hematocrit level, hemoglobin and platelets were sent daily.

At 33 weeks of gestation she was planned for elective cesarean section and she delivered four newborns, one female and three male babies. The placenta was quadriamniotic and quadrichorionic weighing 1300 gms combined. Total blood loss measured about 600 ml. Table 1 shows the details of the four babies at the time of birth (Table1). Liquor of each baby was green and adequate. The intra-operative period was uneventful. The babies were transferred to Neonatal Intensive Care Unit (NICU) for supportive therapy.

**Table 1:** Details of four babies at the time of birth.

Quadruplets	Sex	Weight	presentation	APGAR score	Time of birth
First	Female	1.7 kg	Cephalic	7/10,8/10	9:39 am
Second	Male	1.53kg	Extended breech	7/10,8/10	9:40 am
Third	Male	1.4kg	Cephalic	7/10,8/10	9:41 am
Fourth	Male	1.02kg	Flexed breech	7/10,8/10	9:42 am

On her 1^st^ post operative day, she had chest discomfort and her saturation was not maintained, she was managed conservatively with chest physiotherapy and incentive spirometry. On her second post operative day she had abdominal distension for which she was managed conservatively. She received injection low molecular weight heparin as a prophylaxis for 6 days. Her hospital stay there after was uneventful and she was discharged on 17^th^ post operative day and two babies were given for Kangaroo mother care (KMC). While her two babies were in NICU under supportive care.

## DISCUSSION

The problem of multiple gestation and its management is becoming increasingly frequent due to current methods of treating anovulatory patients.^7-9^ Multiple pregnancy is considered a high risk pregnancy as more complications are observed with the increase in number of fetuses. Higher order pregnancy needs a multidisciplinary approach for the safe transition of a pregnant women to motherhood. Not only obstetrician but early involvement of neonatologists and anesthesiologists for the assessment of such case is essential for a successful obstetrics outcome. Most of higher order pregnancies are associated with severe maternal and perinatal morbidity and mortality. Maternal complications as pre-eclampsia, gestational diabetes mellitus, cardio pulmonary embarrassment, abruptio placentae, incompetent cervix and preterm labour are well documented.^10^ Perinatal complications such as prematurity, congenital anomalies, respiratory distress syndrome, perinatal mortality, twin to twin transfusion syndrome, intraventricular hemorrhage may be present.^11^

In our case, respiratory discomfort was noted, and at 33 weeks of gestation elective cesarean was performed for the same problem. Preterm delivery is a common complication with the mean gestational age at delivery being 35 weeks for twins, 32.2 weeks for triplets and 29.9 weeks in quadruplets.^12,13^ The case reported here also had a preterm delivery with gestational age being 33 weeks. With prematurity two babies were in NICU for supportive care while two babies were given for Kangaroo mother care. In France among 65 quadruplets delivery, the mean gestational age at delivery was 31.2±3 weeks with birth weights of quadruplets ranged from 760 to 2455g with a mean of 1615g.^14,15^ In our case, birthweight ranges from 1.02kg to 1.7kg with mean birth weight of 1412g which is lesser than the above-mentioned data.The low birth weight may be a consequence of premature delivery.

Bed rest, beta- mimetics, progesterons and elective cervical cerclage are done and suggested to prolong the pregnancy.^12,16,17^ The patient was kept in bed rest for a week before delivery. In the reported patient, the quadruplet pregnancy reached the 3^rd^ trimester without prophylactic cervical cerclage. Early hospitalization to plan the management of such case is required for its successful outcome.
